# Tending the ‘monthly flower:’ a qualitative study of menstrual beliefs in Tigray, Ethiopia

**DOI:** 10.1186/s12905-018-0676-z

**Published:** 2018-11-13

**Authors:** L. Lewis Wall, Kibrom Teklay, Alem Desta, Shewaye Belay

**Affiliations:** 1 Department of Anthropology, College of Arts and Sciences, Washington University in St. Louis, Campus Box 1114, One Brookings Drive, St. Louis, MO 63130 USA; 2 Department of Obstetrics & Gynecology, Ayder Comprehensive Specialist Hospital, College of Health Sciences, Mekelle University, Mekelle, Ethiopia; 3 Department of Obstetrics & Gynecology, Washington University in St. Louis, St. Louis, MO USA; 4 MAKIO Consultancy Services, Mekelle, Ethiopia; 5 School of Public Health, College of Health Sciences, Mekelle University, Mekelle, Ethiopia; 6 Department of Microbiology and Parasitology, Institute of Biomedical Sciences, College of Health Sciences, Mekelle University, Mekelle, Ethiopia

**Keywords:** Menstruation, Adolescence, Ethnography, Ethiopia, Menarche, Stigma, Hygiene, Menstrual hygiene management

## Abstract

**Background:**

Menstruation is a universal aspect of human female reproductive life. Management of menstrual flow presents hygiene challenges to girls and women in low-income countries, especially when they first start their periods. As part of a project to improve menstrual hygiene management in the Tigray Region of Ethiopia, we explored the local understanding of menstruation through focus-group discussions and individual interviews.

**Methods:**

A detailed ethnographic survey of menstrual beliefs was carried out through 40 focus group discussions, 64 in-depth key informant interviews, and 16 individual case histories in the Tigray Region of northern Ethiopia. A total of 240 individuals participated in six types of focus groups (pre-menarchal girls, menstruating adolescents, married women of reproductive age, post-menopausal women, adolescent males, and married men). In-depth interviews were also carried out with 80 individuals, including Orthodox Christian priests, imams from the Muslim community, principals of primary and secondary schools, teachers and nurses, as well as menstruating schoolgirls and women. Audio data were transcribed and translated, then broken down into discrete codes using Atlas Ti software (version 7.5.4, Atlas.ti Scientific Software Development Mnbh, Berlin) and further grouped into related families and sub-families based on their content. The results were then synthesized to produce a cohesive narrative concerning menstruation in Tigray.

**Results:**

Recurrent themes identified by participants included descriptions of the biology of menstruation (which were sometimes fanciful); the general unpreparedness of girls for menarche; cultural restrictions imposed by menstruation on females (particularly the stigma of ritual uncleanliness in both Christian and Muslim religious traditions); the prevalence and challenges of unmet menstrual hygiene needs at schools (including lack of access to sanitary pads and the absence of acceptable toilet/washing facilities); and the stigma and shame associated with menstrual hygiene accidents in public.

**Conclusions:**

Changes in the educational system in northern Ethiopia are required to improve student understanding of the biology of menstruation, to foster gender equity, to overcome the barriers to school attendance presented by poor menstrual hygiene management, and to create a society that is more understanding and more accepting of menstruation.

## Background

Periodic menstrual bleeding is an integral component of the human female reproductive cycle, [1] but the way this phenomenon is viewed and the meanings attached to it vary considerably in different societies around the world [2–5]. Most cultures require that menstrual flow be regulated or contained in some fashion. This cultural requirement creates a recurrent challenge to all menstruating girls and women. These menstrual management challenges are often most acute at menarche, because newly-menstruating adolescent girls often lack the knowledge, experience, and self-confidence necessary to deal with the problems of menstrual hygiene [5–15]. Factors that influence menstrual hygiene management include knowledge of reproductive biology; the background beliefs about menstruation prevalent in society; the limitations (if any) that beliefs about menstruation place on female activities; and the ease of access that girls and women have to menstrual management supplies such as commercially-manufactured sanitary pads (either disposable or reusable), tampons, or traditional menstrual management materials obtained from the home environment such as strips of cloth torn from old bedding, skirts, shawls, or other articles of clothing. Particularly in low-income countries, menstrual hygiene management may present a formidable obstacle to the education and social advancement of adolescent girls, who are often confused, embarrassed, and unprepared for the sudden and unexpected appearance of menstruation [12–20].

**Fig. 1:**
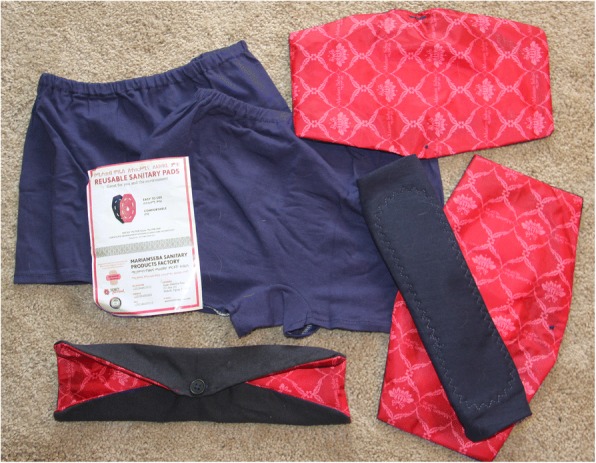
Menstrual hygiene kit distributed by the Dignity Period Project, consisting of 2 pairs of underwear and 4 re-usable menstrual pads. The pads have a waterproof backing; a soft, cotton absorbent lining; and they fasten over the crotch of a pair of underwear using a button. The pads are cleaned by soaking in water, hand-washing with soap, rinsing, and then air-drying in the sun. Ultraviolet radiation from direct sunlight has a bacteriocidal/bacteriostatic effect. Photo by the authors

The Dignity Period Project is a collaborative community development project between Mekelle University (a large Federal university in northern Ethiopia), the Mariam Seba Sanitary Products Factory (a woman-owned social enterprise), and Dignity Period (a not-for-profit public charity based in the United States, www.dignityperiod.org) which seeks to improve knowledge about menstruation in northern Ethiopia and to provide free, locally-produced, environmentally friendly, reusable menstrual hygiene products to schoolgirls in this region. The project hopes to improve the psychosocial environment of Ethiopian girls with respect to menstruation, to reduce school absences from menstruation-related causes, and to stimulate the growth of a viable commercial industry that someday can meet the need for menstrual hygiene supplies in an efficient, locally-sourced, and cost-effective manner. The project provides educational materials about menstruation to girls and boys in Ethiopian schools through a bilingual (English/Tigrigna) educational booklet [21] and on-site, in-school training on menstrual hygiene management. The project also provides schoolgirls with free menstrual hygiene kits containing 2 pairs of underwear and 4 washable, reusable menstrual pads [Fig. 1]. By the end of 2018, the project will have reached over 250,000 students in Tigray and in the contiguous Afar Region of Ethiopia. The present study is part of ongoing efforts to understand menstruation and menstrual hygiene in this part of the country.

## Methods

An ethnographic survey of menstrual beliefs in Tigray, northern Ethiopia, was carried out through focus groups, in-depth interviews, and individual case studies in conjunction with a previously-published community-based survey of this topic [22]. There were 40 focus group discussions, 64 in-depth interviews with key informants, and 16 individual case histories. A total of 322 individuals participated, including 240 people in six different types of focus groups: pre-menarchal girls (*n* = 36), menstruating adolescents (*n* = 48), mature married women of reproductive age (*n* = 48), post-menopausal women (*n* = 48), adolescent males (*n* = 30), and mature married men (*n* = 30). In-depth interviews were also carried out with 80 individuals, including Orthodox Christian priests (*n* = 12), imams from the Muslim community (*n* = 8), secondary school principals (*n* = 12), teachers (*n* = 16; 6 male, 10 female) and nurses (*n* = 16; 5 male, 11 female), as well as menstruating schoolgirls (*n* = 16). Focus groups were run by native speakers of the local language (Tigrigna), except in the Tahtay Adyabo subdistrict, where indigenous speakers of Kunamigna were used as translators for this ethnic group. The sessions were recorded, transcribed, and then translated into English. Audio data were transcribed and translated, then broken down into discrete codes using Atlas Ti software (version 7.5.4, Atlas.ti Scientific Software Development Mnbh, Berlin) and grouped into related families and sub-families based on their content. The results were synthesized by themes to construct a narrative concerning menstruation in Tigray.

The study protocol was reviewed and approved by the institutional review boards at Ayder Comprehensive Specialist Hospital, College of Health Sciences, Mekelle University, Mekelle, Ethiopia, and by the Washington University School of Medicine in St. Louis, MO, USA. Informed consent was obtained prior to each interview or focus group discussion. Study subjects were compensated for their time and participation with 50 Ethiopian Birr (approximately US$2.50 at the time of the study).

## Results

A number of recurrent themes emerged in the course of these interviews and focus-groups. We have organized these themes into four broad categories for the purpose of discussion: the nature and biological function of menstruation; menarche; menstrual restrictions; and menstrual needs, hygiene accidents, and associated stigma.

### The nature and biological function of menstruation

There is universal agreement that menstruation is a normal physiological process linked to reproduction. Most people refer to it as a “gift from God” which permits women to become pregnant and bear children, to give the gift of new life, to become mothers. As one woman said, *“It means the body is ready to reproduce.”* Only the most educated members of our participant population (teachers, nurses, school directors, and a few older students) could provide a reasonably accurate description of the biology of conception and its relationship to menstruation. A typical approximation of these responses was given by one male who noted *“menstrual blood is part of life and the process of making life. It becomes blood if it is not fertilized”—*a recognition that menstruation regularly follows when fertilization does not occur. It is generally understood that menstruation stops during pregnancy.

Menstrual periods are described as occurring monthly (roughly every 21–35 days) with some variability, and bleeding is said to last for roughly 3–7 days. Periods of longer duration or more unpredictable occurrence are regarded as potential reasons to seek medical advice. There is good awareness that certain contraceptive technologies (oral contraceptive pills, medroxyprogesterone acetate injections, etc.) can cause changes in the menstrual cycle. There is also a recognition that menstruation ceases when menopause occurs (biologically around age 50, but commonly described by participants as occurring somewhere between ages 40 and 60). At menopause the potential for becoming pregnant also ceases.

The phenomenon of menstruation has different names in different Ethiopian languages. Menstruation is commonly called the “monthly flower” (in Amharic, *yewor abeba;* in Tigrigna, *werhawi tsigya*). As one Orthodox Christian priest explained it: *“Menstruation is like flowers. Flowering plants finally produce fruit. The same is true of girls after menstruation.”* Another common expression for menstruation in Tigrigna is *nay adetatna* (“[the thing] belonging to our mothers”), which also links it clearly to reproduction. In the Kunamigna language menstruation is called *mara* (“menstruation”) or *ginayaka* (“monthly bleeding from our mothers”). Among the Raya people and others (both Muslim and Christian) it is often called *adef* (“dirty matter”).

As discussed below, menstruation is commonly regarded as ritually polluting. Many women also believe that it is a cleansing process. One menopausal woman remarked *“the blood that comes during menstruation is bad blood and after bleeding the woman makes good blood.”* Another woman said *“It releases bad blood out of our body; it cleanses the system.”* Still another woman remarked “*It voids useless fluids from your body.”* The notion that loss of menstrual blood cleanses or purges the body is common in many other cultures [2, 3].

### Menarche

Males and females both agree that menstruation occurs for the first time in adolescence (roughly between ages 14–18, but in some cases as early as age 7 or 8) and that it heralds the onset of female reproductive capacity like the first “blossoming of a flower.” There is a widely held consensus that the timing of the onset of menstruation varies depending on the girl’s constitution and her living conditions: in the view of many people, good health and good nutrition are linked to an earlier occurrence of menarche.

There is also a common, but not universal, belief (more commonly held by males) that menstruation does not start until a girl has sexual intercourse for the first time. The existence of this false belief presents a potential hazard for girls approaching menarche if they live in families where this belief is held. We know of some girls who have been beaten by their fathers at menarche because they were assumed to be sexually active and misbehaving, when, in fact, they were merely bewildered adolescents having their first menstrual periods. The emotional scars of such experiences are likely to be deep and long-lasting. This belief also may result in some schoolgirls being disparaged by boys as having lost their virginity if they suffer a hygiene accident at school that results in their menstrual status becoming known.

Many Tigrignan girls enter adolescence with little or no knowledge of menstruation. Their first periods are often unexpected, traumatic experiences, as several participants described.

*“It [menarche] happened to me when I was going back to my house from a test I had taken in the eighth grade. I had not been feeling well during the day. In the middle of the road I started to bleed and I was scared. I used a piece of cloth and put it in my underwear. Nobody saw me; but I was very worried and frightened. I didn’t tell my parents.”*

Another girl said “*I went to school and while I was learning suddenly the blood was visible through my uniform. I was shocked and embarrassed. I covered up my uniform with my school bag and went home. I didn’t tell my mother what happened and I immediately went to my room so as not to be seen.”*

An older woman remarked: “*I was in school when I first had my period. When I felt it, I thought that it was urine and I had to go to the bathroom. When I went to the bathroom, I felt the blood. I had no underwear. I had to use the inside of my skirt and wrap it around my body. Then I went back to class and sat on my exercise book. I managed to avoid getting my dress soaked in blood by keeping it away from the blood. I had to wait until all of the other students were gone before I stood up and left the classroom. Then I went to my house and didn’t talk to my parents or siblings. In the meantime, I had to steal my older sister’s underwear and use it. I used dirty, worn-out clothes that were not useful for other purposes. Finally, I told my friend about it. She was my schoolmate. She spoke with our teacher and they bought me underwear and a pad.”* Stories of this kind are common.

### Menstrual restrictions

Numerous cultural restrictions are imposed during menstruation in Tigray. Although girls and women are expected to continue their normal household chores while menstruating, there is a general belief that menstruants are vulnerable and should not do heavy physical labor while on their periods. Hard physical work is thought to increase the bleeding. One woman explained *“A woman’s womb is open during menstruation and if you load yourself with a heavy load, it can cause a problem and too much bleeding.”* The underlying belief appears to be that the uterus is like a sack full of blood and if you strain, more blood comes out. Women probably do notice an increase in bleeding with straining or lifting, particularly if they lack underwear or effective sanitary pads to contain the menstrual flow. On the other hand, there is also the pragmatic realization that the daily work of the household must get done irrespective of what else is happening. For poor women in particular, there is no alternative but to do the work themselves. Economic necessity overrides personal preferences.

Many informants expressed the belief that menstruating women should avoid exposure to direct sunlight. There is a prevalent belief that if a menstruating woman is out in direct sunlight she will develop a debilitating and potentially serious medical condition called *michi*. Thus, to the ritually polluting nature of menstruation is added the belief that being “out” risks serious health consequences to the woman herself—a belief that also keeps the subject of menstruation “in the shadows,” both figuratively and literally.

A more common concern than the development of *michi*, however, is the presence of menstrual cramps and how to manage them. Dysmenorrhea (which is fairly common among adolescents, especially in the first few years after menarche) is often cited as a reason that menstruating girls do not attend school. School absences are also increased if they have difficulty accessing adequate menstrual hygiene supplies. Acetominophen (paracetamol) is frequently used as a treatment for cramps, but it is not always readily available in schools and access to more effective non-steroidal anti-inflammatory drugs such as ibuprofen or naproxen is almost non-existent. Many girls report trying to manage menstrual cramping by taking hot drinks, applying hot water bottles, or resorting to traditional folk-remedies such as rubbing the abdomen with butter to soothe the cramping. Lack of access to effective pain medications for menstrual cramps while at school was cited as a common problem that adversely impacted school attendance and learning.

The intake of certain foods and fluids is thought by many to influence menstrual flow, but these beliefs are inconsistent and often contradictory, reflecting an uncertain understanding how they might work. (In fact, there is no physiological reason to believe that food or fluid intake would affect the quantity or duration of menstrual flow). Many people say menstruating girls should take hot drinks while menstruating, perhaps for their soothing qualities; but others deny this and say that hot drinks only serve to increase the amount of menstrual flow and that menstruants should take only cooling drinks instead. In many cultures there is a belief that “cooling” the blood leads to clot formation and sluggish menstrual flow, whereas heat is thought to increase menstrual circulation [2]. Something conceptually similar to this may be in play here. Most respondents believe that women should take “good” (i.e., nourishing) food (many also mentioned iron supplements, perhaps a reflection of public health education pertaining to anemia) and stated that girls and women should avoid overwork while menstruating in order to conserve their strength.

Unlike some parts of the world, there do not appear to be restrictions in Tigray on the ability of menstruating women to prepare food (assuming they keep themselves clean and wash regularly). Only one or two men said they would prefer *not* to eat food cooked by a menstruating woman. A more typical response was offered by one Orthodox priest (a married man), who, when asked whether or not he would eat food cooked by his wife if she was menstruating, said somewhat incredulously: *“Well, who else would prepare it for me?”* Cooking is a responsibility that women are expected to assume in the household and this is not abrogated by menstruation.

Nonetheless, menstruation is foreign to male physiological experience and is not usually discussed openly. There is a general male wariness about menstrual blood. Many informants reported that diseases could be transmitted through menstrual blood (including genital infections, HIV, hepatitis, and even tetanus). One said: *“If women don’t dispose of hygienic materials properly and if it touches body parts that may be sensitive to disease, it may cause disease.”* Such beliefs about the potential infectiousness of blood have likely been increased by HIV/AIDS awareness campaigns in Ethiopia.

When asked if it was permissible to have sexual relations with a woman while she was menstruating, there was generally strong disapproval of this practice by both males and females. Although some people believed that such intercourse would result in pregnancy, almost all respondents voiced cultural, religious, and aesthetic disapproval of having intercourse with a menstruating woman. One woman explicitly likened this to incest. Referring to menstruation as *“[the thing] of our mothers,*” she said *“If a man sleeps with his wife when she is on her menstrual period it is like he was sleeping with his mother,”*--a very strong aversion indeed! Orthodox priests and Muslim imams both said that sex was not permitted during menses. *“I won’t have sex with a menstruating girl,”* one young man reported, *“because her blood may contaminate me.”* Another young man said *“It is forbidden to have sex with a menstruating girl in our tradition. Besides this,”* he added, *“girls smell bad during their menstrual periods.”* A specific aesthetic objection was thereby added to the general cultural disapproval of this practice.

There is a strong cultural tradition among both Orthodox Christians and Muslims that menstruating girls and women are ritually impure and should be excluded from religious activities. Menstruants are prohibited from entering the mosque or the church; rather, they should remain outside and pray by themselves. This largely appears to be a self-imposed restriction, however; we are not aware of any attempts to check the menstrual status of women at sites of religious worship. As such, a girl’s menstrual status is a matter between her and God, but many people seem to feel that God Himself would be personally offended by a menstruating girl or woman who comes into His holy presence. One pre-menarchal girl declared with certainty: *“She [a menstruating girl] can’t fast or pray in a church for it is useless and not accepted by God.”* Many people also expressed the belief that menstruants should not touch sacred objects such as the Bible, a cross, holy water, or the Koran. Such beliefs about menstruation and ritual pollution put women at a serious disadvantage in a society where religious influences remain very strong and permeate many aspects of the culture.

### Hygiene needs, menstrual pads, and menstrual stigma

How to manage menstrual hygiene is an omnipresent concern for females, particularly for poor girls and women in rural areas where living standards may be often marginal. The embarrassment and shame that result from a public menstrual hygiene accident can be overwhelming for the girl who experiences it, particularly during her vulnerable early adolescent years. We heard many reports of schoolgirls who were taunted by boys (and sometimes girls) who ran after them, shouting and making fun of them if they had visible menstrual staining on their clothes at school. The mortification that results from such public shaming can be severe. One girl reported an incident in her school in which a very bright female student was called to the front of the classroom to write on the blackboard (an everyday experience in Ethiopian schools) only to realize that the entire class was staring at her backside. To her horror she discovered that the seat of her dress (which was turned directly to the class as she wrote on the blackboard) was soaked in menstrual blood. She was so ashamed that she left the classroom immediately and decided to change schools rather than return to the scene of her humiliation. Other girls who have experienced such accidents stop coming to school while on their periods; some simply drop out altogether.

One older woman reported concerning her own girlhood: *It was difficult back in the day* [when you were on your period.] *You sat down and stayed in one place. If you were making coffee* [a common and important Ethiopian social ritual for guests]*, you would prepare everything but not move at all. You would be careful not to get blood on your dress. You just covered yourself and were self-conscious. You would be especially careful if there was a guest or a boy who might see you.*

Another woman said: *“Back then there were no things like underwear and sanitary pads. I lived in a village and we didn’t know about it. It was very difficult to socialize without having underwear. It was common to miss two or three days [of school each month]. I used to miss class. I menstruated at 15 or 16 but there was no underwear or sanitary pads. We wore traditional [white] dresses and it was not easy to control menstruation. We were farmers. If I was seen bleeding in class the students would make fun of me.”* The rest of her focus group chimed in with enthusiastic support, shouting *“That is it! She has said it!”* Her comments touched a “social nerve” that was felt by all the women present.

Many teachers reported that poor girls routinely skipped class during their menstrual periods for fear of being publicly exposed to such shaming. One adolescent boy remarked, *“I don’t think girls ever stand up without looking at their seats because they know what will happen if students see blood.”* One adolescent schoolgirl said *“At school there is not any kind of support* [for menstruation]. *If we bleed, we have to go home because we have financial problems* [and cannot afford to buy menstrual pads]*.”* The fear of exposure of their menstruating state due to an accident of hygiene is constantly on the minds of girls and keeps them from concentrating on their lessons. One informant said, *“A menstruating girl is afraid to go to school and when in class she cannot pay attention due to fear of menstrual staining.”* Another girl said, poignantly, *“My menstrual pad fell out while I was playing football [soccer] at school. All the students laughed at me and shouted at me and I missed school for three days.”* The stress of participating in required vigorous physical activity while trying to wear a menstrual pad with no underwear to support it was extremely stressful and several women reported similar incidents in their lives.

One of the most common obstacles to school attendance is lack of adequate menstrual hygiene supplies. Commercially produced, disposable pads are often not accessible, but even when they are available, they are too expensive for poor rural woman to buy. Proper disposal of such pads is also problematic. The price of a package of disposable menstrual pads was stated as 18–25 Birr (roughly $US 0.80 to $US 1.10) per month, but as several women stated, *“It is very expensive. We are poor”* and *“For a poor woman, it’s difficult even to afford one pair of underwear.”* One woman remarked, *“It’s expensive, let alone 18 Birr! Even 8 Birr [$US 0.35] is expensive!”* Another said, *“Some can’t even afford three or four Birr”* [~$US 0.18]. A Muslim iman with a large family lamented, *“They cost 18 Birr—if you have three daughters it will be especially difficult.”* For him, menstruation was a familial financial catastrophe. The alternative to using commercially-produced sanitary pads is to make do with strips of cloth torn from old dresses, pieces of old mattresses, or whatever absorbent materials may be at hand, but such remedies are often not effective. As one teacher summarized the problem, *“Most of the girls do not attend class* [when they are menstruating] *since they don’t have menstrual pads or even clean pieces of cloth.”*

There is a general consensus that good hygiene is important. The most common view is that girls and women should wash themselves several times per day when they are menstruating and that this is particularly important if they don’t have menstrual pads; but scarcity of water is a constant, pervasive problem throughout Tigray, particularly in schools. Women in rural areas may have to carry water several miles from the nearest water-source in order to have a supply at home, which makes water a precious household commodity. Lack of water for washing (let alone drinking) is very common, particularly in rural schools. Many schools have no source of running water on site and the only water available is whatever students can carry from home in containers for their daily use. There is also a belief (more notable in rural areas and among older women) that washing or bathing during menstruation increases the flow of blood. In the past, many girls were actually forbidden to wash while menstruating, which undoubtedly only increased their discomfort. These beliefs may be intertwined with one another.

**Fig. 2:**
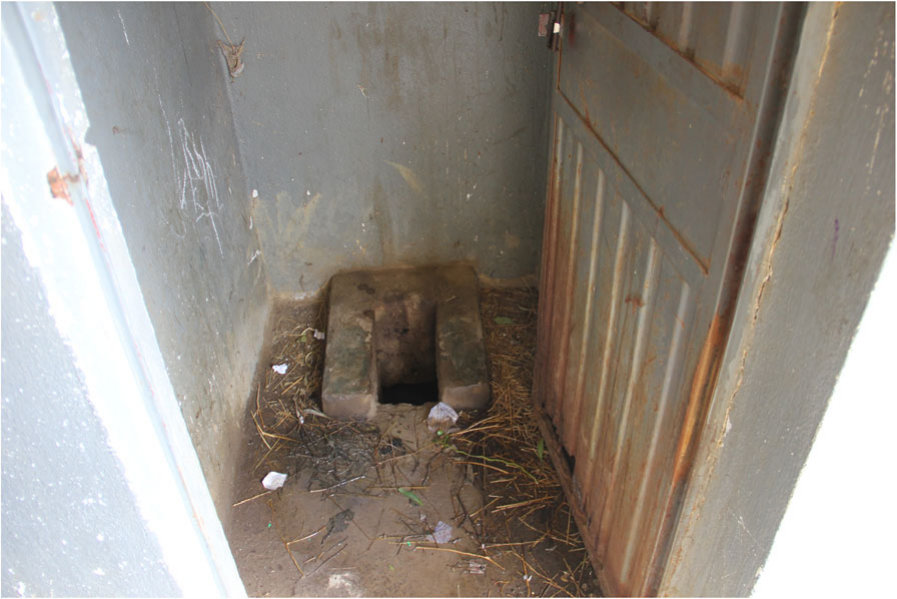
Typically inadequate toilet facilities at a secondary school in rural Tigray. Photo by the authors

There was general agreement that schools should furnish menstrual pads to girls and that schools should also have medications for menstrual cramps available for student use. Both strategies were felt to be beneficial for improving school attendance. There was also general agreement that toilet facilities at schools were inadequate—especially for girls---and needed much improvement. This is readily demonstrable in almost any school in the region [Fig. 2]. There was agreement that girls’ toilets should be well separated from those used by males to ensure better privacy, especially when dealing with intimate issues such as washing and changing menstrual pads during the day. When asked in a focus group discussion what should be provided in school latrines, one woman stated forcefully: *“I would build a latrine with a shower and toilet inside. I would have well-made menstrual pads and soap! And it would have a continuous supply of water and soap!” [much laughter and cheering from the group].* Achieving this ideal seemed hopelessly remote, given the local economic circumstances, but nonetheless such improvements were greatly desired, and it was thought that such facilities would contribute substantially to improved gender equity in the Tigray Region.

## Discussion: Menstrual hygiene Management in Context

In recent years menarche and menstruation increasingly have become recognized as important public health issues with particular salience for the health and psychosocial well-being of adolescent girls [13–16, 20, 22–24]. The increasing attention paid to these issues has produced several qualitative studies from East Africa (Kenya, Tanzania, Uganda, and Ethiopia) which document the impact that lack of adequate resources for coping with menstrual hygiene has on adolescent girls [6, 12, 19, 22–34]. Menstrual hygiene management is difficult and inadequate throughout Ethiopia (http://www.pma2020.org/sites/default/files/ETR5-MHM%20brief-v1-2017-08-14_0.pdf). Our study confirms and enlarges the qualitative research on menstruation, with specific reference to northern Ethiopia.

The challenges Tigrayan schoolgirls face in managing their menses is rooted in a general cultural reluctance to discuss the subject of menstruation, a problem which is made worse by inadequate scientific education concerning sexuality and human reproductive biology. The failure to discuss menstruation at home (which is often grounded in parental discomfort with the topic, personal embarrassment, and a denial of their daughters’ impending sexual awakening) means that many (perhaps most) girls arrive at menarche unprepared for the experience of menstruation. This phenomenon is certainly not limited to Tigray [4–20, 30, 31]. When their menstrual bleeding first begins—often unexpectedly—girls may be frightened, embarrassed, and suffer unnecessary psycho-social trauma. Some people in this region believe that menstruation does not begin until a girl has sexual relations [22], which may precipitate unfounded, scurrilous rumors about sexual misbehavior and provoke baseless family conflict. Not knowing what is really happening during their menses, many girls are worried, fearful, and go to great lengths to hide their bleeding from others, especially from family members. For these girls, containing the menstrual flow is a major, anxiety-ridden preoccupation while they are on their periods. This task is made more difficult by lack of access to satisfactory menstrual management materials, which are either unavailable, too expensive, or have inadequate absorbent capacity. Male family members who control financial resources often do not understand the importance of this aspect of their daughters’ lives because of ignorance or benign neglect [35].

The resultant anxiety over possible menstrual hygiene accidents at school or elsewhere in public is often so high that girls stay home while menstruating and fall behind in their studies. The stigma associated with menstruation is increased by negative religious attitudes that categorize menstruating women as ritually unclean, as well as by thoughtless, mean, and sometimes cruel taunting and teasing behavior by fellow students (especially adolescent boys) when menstrual hygiene accidents occur. The school environment is perceived as difficult for menstruating girls due to the social pressures surrounding this normal physiological process, and this is worsened by pitifully inadequate toileting facilities, poorly constructed latrines, lack of access to soap and water for cleaning, lack of menstrual pads when menstruation begins unexpectedly, and lack of basic medications to help with menstrual cramps when these occur. Our findings from Tigray confirm the presence in that region of the problems so commonly reported elsewhere [36]. The Kenyan comment that “the girl with her period is the one to hang her head” [6] applies equally to the Tigray Region of northern Ethiopia. We have not, however, found evidence thus far that Tigrayan girls trade sexual favors for access to menstrual hygiene supplies as has been reported in Kenya [29]. This should be a topic for further investigation.

Meeting the challenges surrounding menstrual hygiene requires a multilateral approach. There is a great need for better instruction in the science of human reproductive biology, including the female hormonal cycle and how it relates to fertility and menstruation. A strong understanding of basic human reproductive biology would go far towards creating a more compassionate attitude towards menstruation. Cultural attitudes towards menstruation in Tigray are strongly influenced by religious perspectives on menstruation that developed centuries before the science underlying the female reproductive cycle was understood. A broader understanding of the biology of menstruation throughout the general population could open the way for more nuanced theological discussions of this and related topics.

In addition, there is a great need for improving the physical environment of Tigrayan schools, especially as it relates to hygiene. In many places latrines are substandard, even unsafe (sometimes located well away from the school grounds and unprotected by fencing that would keep interlopers away). Boys’ latrines should be segregated from girls’ latrines and there should be enough room and enough privacy within girls’ latrines to allow easy changing of menstrual pads and attention to basic washing and cleaning [36]. The provision of fresh water is a particular challenge in Tigray, where drought is common and on-site water sources often do not exist. Each school should make sure that it has an acceptable, functioning gender office which can supply sanitary pads on an emergency basis, basic medication for the relief of menstrual cramps, and a place for girls to rest if necessary. The Tigray Regional Educational Bureau is taking steps to make this happen.

By itself, improved education about menstruation appears to have a positive impact on menstrually-related absences by adolescent schoolgirls [37]. It seems likely that provision of menstrual hygiene supplies to students also has a positive impact on school attendance by girls, although further research in this area is needed to solidify this perception [37–39]. In light of the fact that menstruation is an unavoidable experience for girls in low-resource countries, effective programs to lower the barrier this presents to school attendance and their further education is highly desirable. Strong evidence demonstrates that further education improves health and family outcomes, lowers child mortality, and produces positive economic benefits [40, 41]. These are all valuable public policy goals. A recalibration of school and community values to make menstrual hygiene a higher priority than it is at present will be necessary to bring this about.

## Conclusion

Changes in the educational system are required to improve the understanding of menstruation by all students, male and female, to foster gender equity and to create a society more understanding and accepting of menstruation.
